# Design for Fe-high Mn alloy with an improved combination of strength and ductility

**DOI:** 10.1038/s41598-017-03862-y

**Published:** 2017-06-15

**Authors:** Seung-Joon Lee, Jeongho Han, Sukjin Lee, Seok-Hyeon Kang, Sang-Min Lee, Young-Kook Lee

**Affiliations:** 1 Department of Materials Science and Engineering, Yonsei University, Seoul, 03722 Republic of Korea; 2 Joining and Welding Research Institute, Osaka University, 11-1, Osaka, 567-0047 Japan; 3 Department of Materials Science and Engineering, Chungnam National University, Daejeon, 34134 Republic of Korea

## Abstract

Recently, Fe-Mn twinning-induced plasticity steels with an austenite phase have been the course of great interest due to their excellent combination of tensile strength and ductility, which carbon steels have never been able to attain. Nevertheless, twinning-induced plasticity steels also exhibit a trade-off between strength and ductility, a longstanding dilemma for physical metallurgists, when fabricated based on the two alloy design parameters of stacking fault energy and grain size. Therefore, we investigated the tensile properties of three Fe-Mn austenitic steels with similar stacking fault energy and grain size, but different carbon concentrations. Surprisingly, when carbon concentration increased, both strength and ductility significantly improved. This indicates that the addition of carbon resulted in a proportionality between strength and ductility, instead of a trade-off between those characteristics. This new design parameter, C concentration, should be considered as a design parameter to endow Fe-Mn twinning-induced plasticity steel with a better combination of strength and ductility.

## Introduction

To make a safer and more energy-efficient society, one of physical metallurgists’ dreams is to create an alloy with a proportional relationship between its strength and ductility. Even though this sounds to be the reverse of the plan of Mother Nature, for the sake of this dream many attempts have been made over a long period of time. However, traditional strengthening mechanisms, such as grain refinement^1, 2^, strain hardening^3^, and dispersion hardening^4^, have been found to result in a reduction of the ductility; this phenomenon is referred to as the strength-ductility trade-off ^5, 6^. Therefore, it has become inevitable to develop new strengthening mechanisms without strength-ductility trade-off. Fortunately, in recent days, some advances have been made, primarily in the field of non-ferrous materials, by adopting a bimodal-grained structure^7^, a hierarchical structure with nanoscaled grains^8^, and a linearly graded nanotwinned structure^6, 9^.

For ferrous materials, Fe-high Mn twinning-induced plasticity (TWIP) steel with a single *fcc γ* austenite phase has attracted significant attention because of its high ultimate tensile strength (UTS) (>700 MPa) and high uniform elongation (UE) (>50%)^10^, which carbon steels with a *bcc*-based matrix cannot achieve. These superior tensile properties are due to the high strain-hardening rate (SHR), which is caused primarily by active mechanical twinning in *γ* austenite with low stacking-fault energy (SFE). Mechanical twins sub-divide grains during plastic deformation and act as obstacles to the dislocation movement^10, 11^. Mechanical twinning is greatly influenced by both grain size^12^ and chemical composition^13^ and temperature-dependent SFE^14^. For example, when the SFE value is less than ~20 mJ m^−2^, the *ε* martensitic transformation usually occurs; when the SFE value is between ~20 and 50 mJ m^−2^, mechanical twinning occurs^10, 11^; when the SFE value is over ~50 mJ m^−2^, dislocation hardening takes place without *ε* martensitic transformation or mechanical twinning.

The effect of grain size on mechanical twinning in Fe-high Mn steels has been extensively investigated^1, 2, 12^. Gutierrez-Urrutia *et al*.^2^ reported that mechanical twinning was suppressed by grain refinement in Fe-22Mn-0.6 C (wt.%) TWIP steel due to the increase in critical twinning stress. Kang *et al*.^12^ reported that grain refinement in TWIP steel increases the back stress of dislocations on the slip planes: the high back stress narrows the width of the stacking faults so that cross slip of dislocations is facilitated; the interaction between partial dislocations required for mechanical twinning is reduced, resulting in inactive twinning. Accordingly, until now the alloy design of TWIP steel has been implicitly performed to explore optimal chemical composition and grain size, which can be used to achieve both a low SFE value ranging from ~20 to 50 mJ m^−2^ at room temperature for active mechanical twinning^14, 15^ and the high *γ* stability necessary to avoid strain-induced martensitic transformation.

However, when designed based only on SFE and grain size, this promising TWIP steel also exhibits the strength-ductility trade-off^10^. Therefore, we here introduce another design parameter of TWIP steel that enhances the mechanical twinning even under the similar conditions of SFE and grain size, resulting in an improved combination of strength and ductility.

## Results and Discussion

### Tensile behavior of TWIP steels

**Figure 1:**
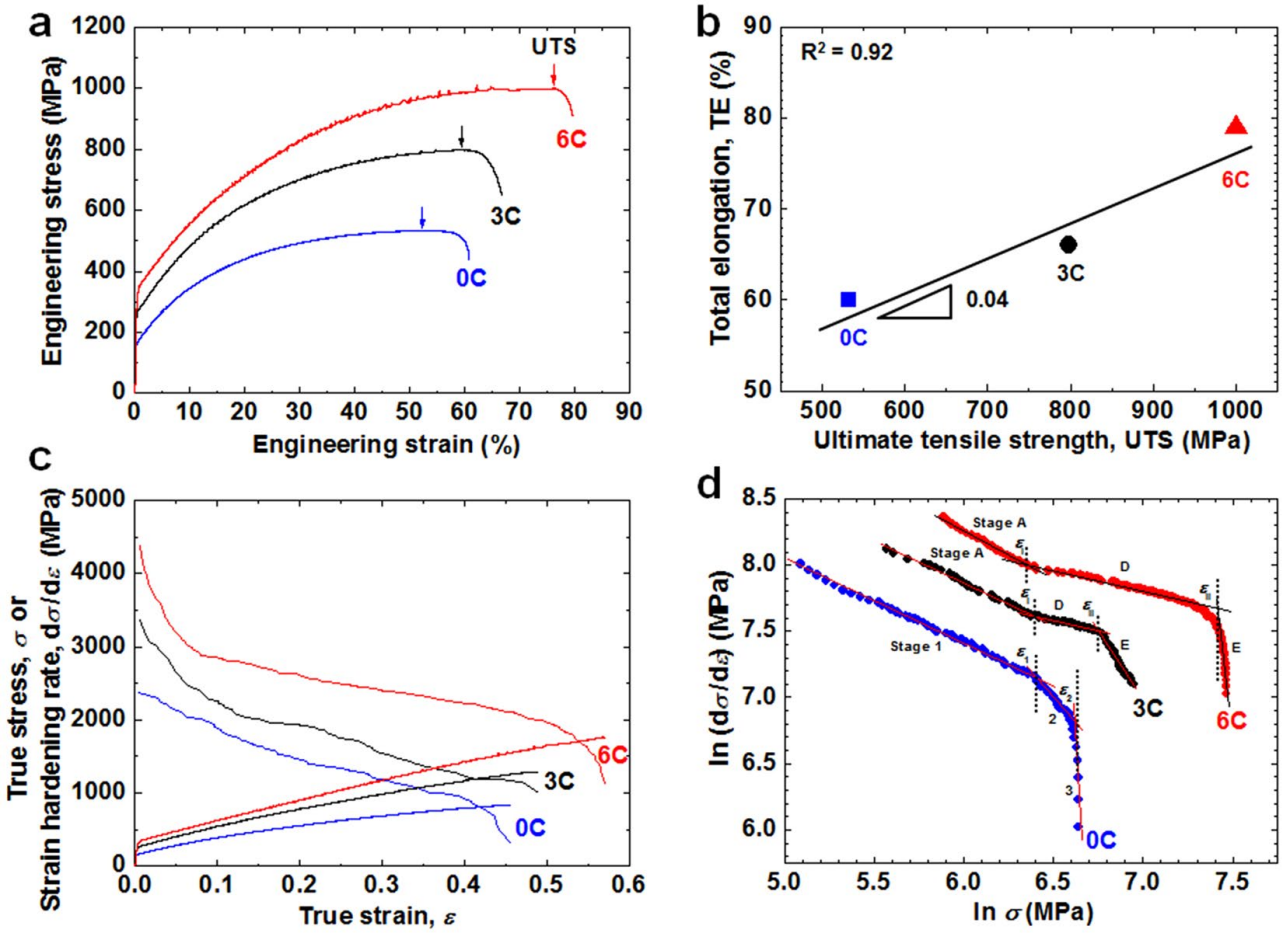
Tensile properties of Fe-high Mn steels (0C, 3C and 6C steels). (**a**) Engineering stress-strain curves, **(b)** total elongation (TE) *vs*. ultimate tensile strength (UTS) plot, **(c)** true stress-strain (*σ* − *ε*) and strain-hardening rate (SHR, d*σ*/d*ε*) curves and **(d)** ln (d*σ*/d*ε*) - ln *σ* plots. All tensile tests were performed at room temperature with an initial strain rate of 1 × 10^−4^ s^−1^.

Figure 1a depicts engineering stress-strain curves of annealed Fe-31Mn, Fe-29Mn-0.3C and Fe-25Mn-0.6C (wt.%) steels. All specimens had a single *γ* phase with similar grain size (~9 μm) and SFE value (~44 mJ m^−2^). Hereafter, these three steels are referred to as 0C, 3C and 6C steels. The 0C, 3C and 6C steels revealed continuous yielding; their yield strength (YS) values were 160, 258 and 312 MPa, respectively (Fig. 1a and Supplementary Table 2). Bouaziz *et al*.^16^ suggested the following empirical equation of YS as a function of chemical composition in weight percent for austenitic Fe-high Mn-C steels:

1 $${\rm{YS}}\,({\rm{MPa}})=228+187{\rm{C}}-2{\rm{Mn}}$$

Eq. (1) indicates that the reason that the YS of the 0.6C steel is higher than that of the 0C steel is primarily the solid solution hardening of C. Ghasri-Khouzani and McDermid^17^ investigated the effect of C concentration on the YS of austenitic Fe-22Mn-(0.2–0.6)C (wt.%) steels with an average grain size of ~110 μm. The YS values rose from 172 to 265 MPa with increasing C concentration from 0.2 to 0.6 wt.%, corresponding to a slope of 24.2 MPa per 0.1 wt.% C. The YS of the three steels used in the present study also increased by 24.1 MPa per 0.1 wt.% C. This means that the YS of the present steels is significantly influenced by the solution hardening of C in *γ* austenite^17^.

Both UTS and total elongation (TE) also improved from 532 to 1000 MPa and from 60 to 79%, respectively, when C concentration increased from 0.002 to 0.62 wt.%. Accordingly, and surprisingly, the addition of C gave rise to a proportionality between the strength and the elongation, as shown in Fig. 1b. This means that the tensile properties of the present austenitic steels with similar grain size and SFE value are also greatly improved by C concentration. It was realized that C concentration, as well as SFE and grain size, is another important design parameter. The improvements of both UTS and TE are most likely due to the enhancement of SHR via the addition of C. As can be seen in Fig. 1c, the higher the C concentration is, the higher the value of SHR will be for the entire true strain range. To investigate the cause of this beneficial effect of C on the SHR, modified Crussard-Jaoul (C-J) analysis^18^, based on the Swift equation^19^, was performed using both true stress (*σ*) - strain (*ε*) and SHR (d*σ*/d*ε*) curves of the three steels (Fig. 1c). The modified C-J plot, *viz*. the ln (d*σ*/d*ε*) - ln *σ* plot, of each steel reveals three distinct stages based on the slope change (Fig. 1d). It is generally known that TWIP steels reveal the following five SHR stages^20^: dynamic recovery of dislocations and no mechanical twinning (stage A), active primary mechanical twinning (stage B), inactive primary mechanical twinning (stage C), active secondary mechanical twinning as well as less active primary twinning (stage D) and thicker twin bundle formation (stage E). However, the SHR stages may merge or disappear, depending on chemical composition, grain size and SFE^12, 20, 21^.

### Microstructural evolution and deformation mechanisms

**Figure 2:**
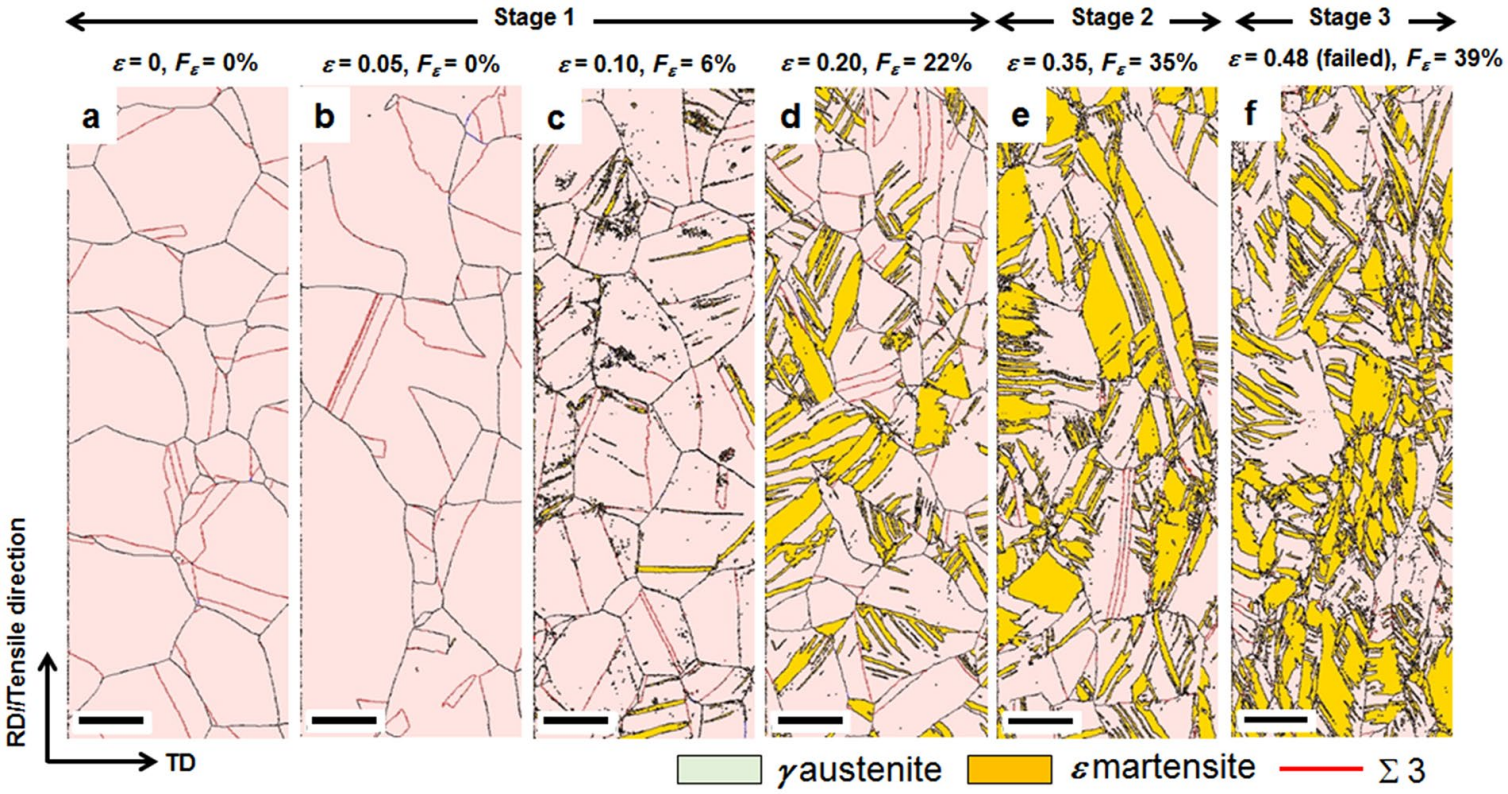
EBSD phase maps showing microstructural evolution with true strain in 0C steel. The strains are (**a**) 0, (**b**) 0.05, (**c**) 0.10, (**d**) 0.20, (**e**) 0.35 and (**f**) 0.48 (failed specimen). RD and TD: rolling and transverse directions, respectively. *F*_*ε*_: the fraction of *ε* martensite. Scale bar, 10 μm.

Accordingly, the microstructure at each stage of the modified C-J plot was observed using an electron backscatter diffractometer (EBSD). Tensile specimens of the 0 C steel with three stages divided into two critical strains (*ε*_*1*_ = 0.28 and *ε*_*2*_ = 0.41) were deformed with different strains and their microstructures were observed (Fig. 2). When *ε* was 0.05 (Fig. 2b), there were neither mechanical twins nor martensite. However, when *ε* increased to 0.10 (Fig. 2c), a small amount of thin *ε* martensite plates formed primarily at the grain boundaries. When *ε* reached 0.20 (Fig. 2d), the fraction of *ε* martensite significantly increased to 22%, and *ε* plates with different crystallographic variants formed within a single *γ* grain. However, the slope of stage 1 was almost unchanged in spite of the formation of *ε* martensite (Fig. 1d). This is not in accordance with the previous result, in which the slope of the modified C-J plot increased due to the formation of *ε* martensite in Fe-18Mn-0.6C-3Si (wt.%) TWIP steel^22^. The insignificant hardening effect of *ε* martensite in the 0C steel can most likely be attributed to the negligible solution hardening due to the absence of C^23^.

When *ε* became 0.35, corresponding to stage 2 (Fig. 2e), the fraction of *ε* martensite increased further to ~35%, and *ε* martensite plates coalesced and became thicker. Accordingly, the reason that the slope of stage 2 decreased despite the increase in the fraction of *ε* martensite (Fig. 1c) is thought to be the coalescence of the *ε* plates. This indicates that the SHR of the 0C steel is primarily influenced by the obstruction of thin *ε* martensite plates of the dislocation slip in the *γ* matrix, not by the fraction of unhardened *ε* martensite.

When the specimen failed (Fig. 2f), the coalescence of *ε* martensite plates proceeded further without a great increase in the fraction of *ε* martensite (~39%). This resulted in a rapid reduction in the slope of stage 3 (Fig. 1d). Meanwhile, mechanical twins were not observed in any of the stages. This implies that the deformation mode of the 0C steel is transformation-induced plasticity (TRIP). Therefore, the stages in the modified C-J plot of the 0C steel are denoted as 1, 2 and 3, instead of the denotations of A, B and C used in the case of the 3C and 6 TWIP steels.

**Figure 3:**
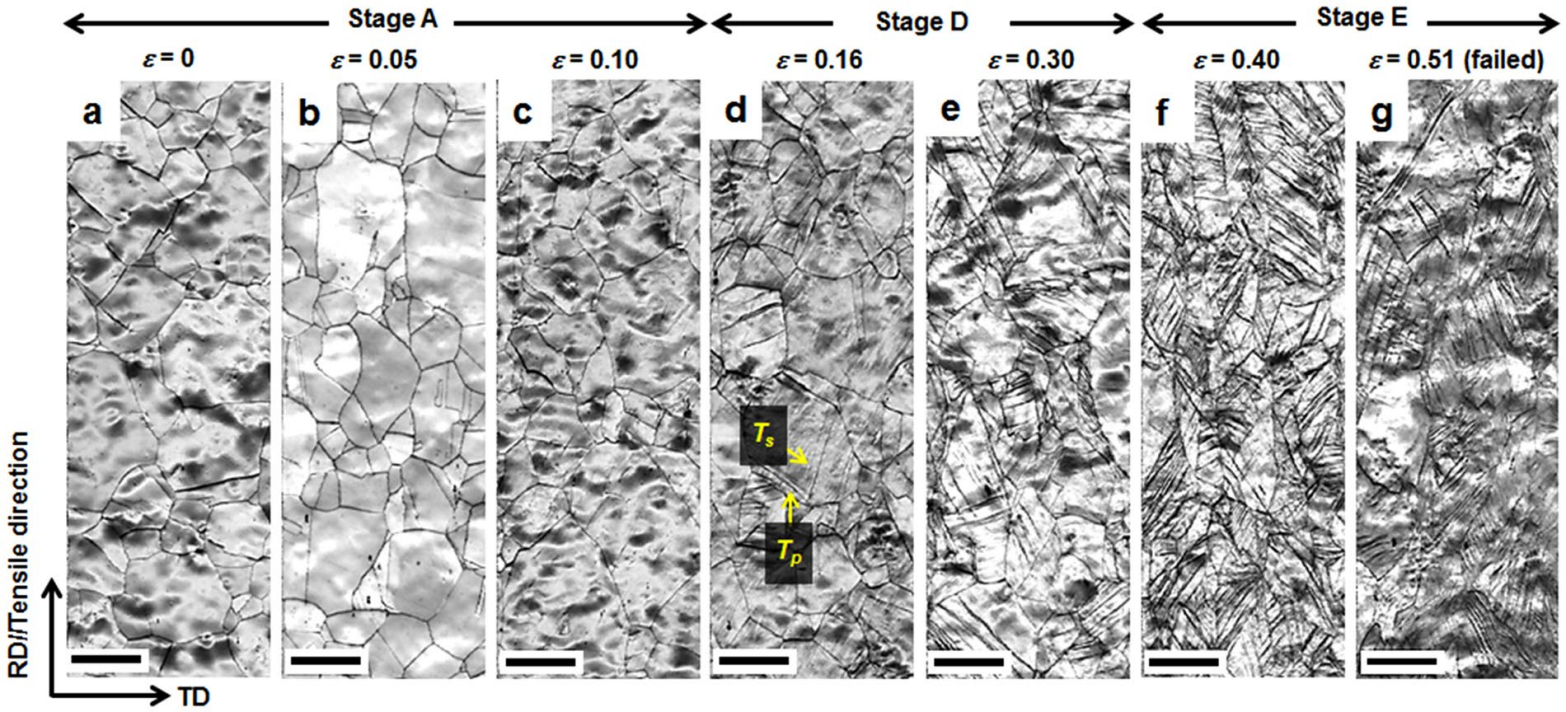
EBSD image quality maps showing microstructural evolution with true strain in 3C steel. The strains are (**a**) 0, (**b**) 0.05, (**c**) 0.10, (**d**) 0.16, (**e**) 0.30, (**f**) 0.40 and (**g**) 0.51 (failed specimen). RD and TD: rolling and transverse directions, respectively. *T*_*p*_ and *T*_*s*_: primary and secondary mechanical twins, respectively. Scale bar, 10 μm.

The microstructure at each stage in the modified C-J plots of both the 3C and 6C steels was also observed using EBSD. The 3C steel exhibited three stages divided into two critical strains (*ε*_*I*_ = 0.13 and *ε*_*II*_ = 0.32). When *ε* was 0.10, corresponding to stage A, neither *ε* martensite nor mechanical twins were observed (Fig. 3c). However, when *ε* reached 0.16, both primary (*T*_*p*_) and secondary twins (*T*_*s*_) were observed (Fig. 3d); their density gradually increased until *ε* = 0.3 (Fig. 3e). This twinning behavior, corresponding to stage D, is thought to increase the slope between *ε*_*I*_ and *ε*_*II*_ (Fig. 1d). Although the fraction of twins increased further after *ε*_*II*_ (Fig. 3f and g), the slope of the modified C-J plot decreased again, most likely due to the thickening of the twin bundles, corresponding to stage E^20^.

**Figure 4:**
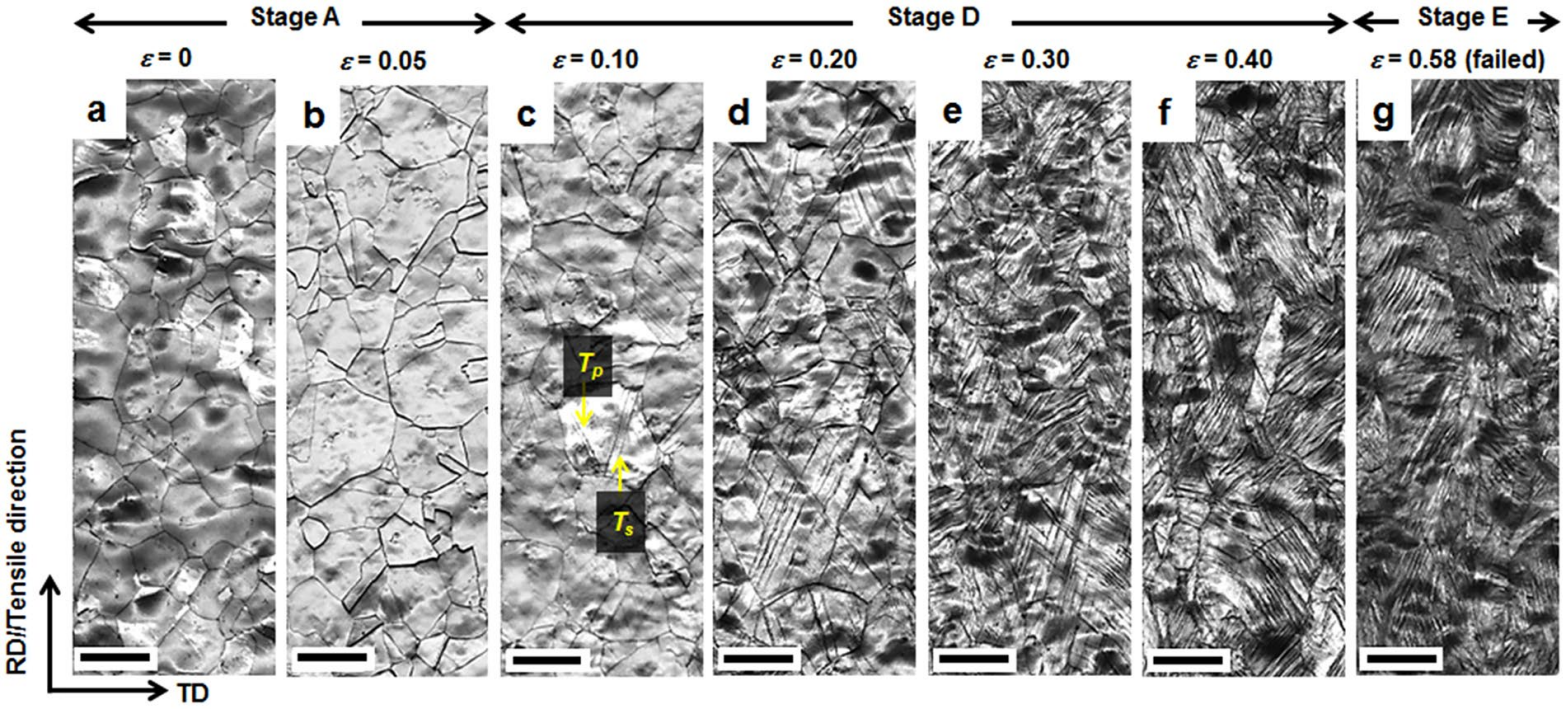
EBSD image quality maps showing microstructural evolution with true strain in 6C steel. The strains are (**a**) 0, (**b**) 0.05, (**c**) 0.10, (**d**) 0.20, (**e**) 0.30, (**f**) 0.40 and (**g**) 0.58 (failed specimens). RD and TD: rolling and transverse directions, respectively. *T*_*p*_ and *T*_*s*_: primary and secondary mechanical twins, respectively. Scale bar, 10 μm.

**Figure 5:**
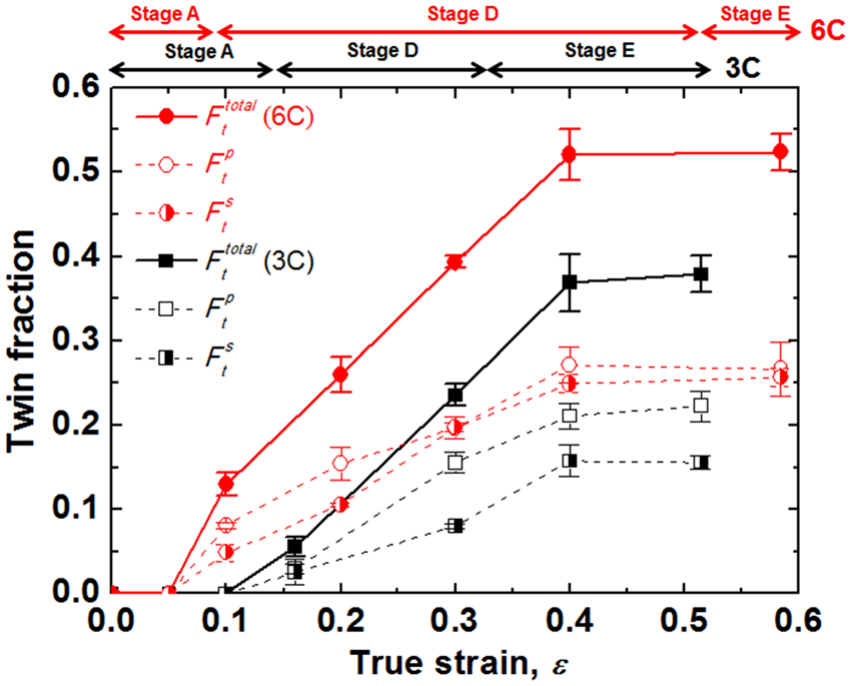
Variations in the fraction of total $({F}_{{t}}^{{total}})$, primary $({F}_{t}^{p})$ and secondary $({F}_{t}^{s})$ mechanical twins with true strain in 3C and 6C steels.

Meanwhile, the 6C steel also revealed three stages divided into two critical strains (*ε*_*I*_ = 0.08 and *ε*_*II*_ = 0.50). When *ε* was below 0.05, corresponding to stage A, mechanical twins were rarely observed (Fig. 4a and b). The 0.1-strained specimen started to exhibit *T*_*s*_ alongside *T*_*p*_ (Fig. 4c). The fractions of both *T*_*p*_ and *T*_*s*_ continued to increase until *ε* = 0.40 (Fig. 4d–f). Accordingly, the increase in the slope between *ε*_*I*_ and *ε*_*II*_ (Fig. 1c) was ascribed to concurrent primary and secondary twinning, corresponding to stage D. The decrease in the slope of stage E is due to the thickening of the twin bundles. The fractions of mechanical twins in both the 3C and 6C steels are plotted as a function of *ε* in Fig. 5. It is obvious that the increase in C concentration triggered mechanical twinning earlier, and generated a higher twin fraction at the same true strain, resulting in the higher SHR.

The aforementioned SHR behavior and microstructural observation gave rise to the important result that the deformation mode (TRIP or TWIP) can change according to the C concentration as well as both SFE^15^ and grain size^1^. This confirms that C concentration should be considered together with SFE and grain size for the alloy design of TWIP steel.

### Critical resolved shear stresses for *ε* martensitic transformation and mechanical twinning

To investigate the reason that C changes the deformation mode, critical resolved shear stresses (CRSS) for *ε* martensitic transformation and for mechanical twinning in the present three steels were evaluated. The influence of the applied stress on the martensitic transformation was investigated through the following thermodynamic calculation^24–26^:

2 $${\rm{\Delta }}{G}_{{M}_{s}}^{{\gamma }\to {\varepsilon }}={\rm{\Delta }}{G}_{T}^{{\gamma }\to {\varepsilon }}+{{\tau }}_{{\varepsilon }}s{V}_{m}$$

where ${\rm{\Delta }}{G}_{{M}_{s}}^{{\gamma }\to {\varepsilon }}$ and ${\rm{\Delta }}{G}_{T}^{{\gamma }\to {\varepsilon }}$ are the differences in chemical Gibbs free energy between the *γ* and *ε* phases at the martensite start (*M*_*s*_) temperature and at the deformation temperature (*T*), respectively_._ *τ*_*ε*_ is the CRSS for the onset of *γ* to *ε* martensitic transformation, *s* is the shear strain for *ε* martensitic transformation (0.353)^27^, and *V*_*m*_ is the molar volume of the *γ* phase (7.074 × 10^−6^ m^3^ mol^−1^)^28^. Therefore, the term *τ*_*ε*_ *sV*_*m*_ indicates the mechanical driving force.

Using the empirical *M*_*s*_ equation for the *γ* to *ε* martensitic transformation^29, 30^ to calculate the ${\rm{\Delta }}{G}_{{M}_{s}}^{{\gamma }\to {\varepsilon }}$ value, the *M*_*s*_ temperatures of the 0C, 3C and 6C steels were found to be 232.5, 121.9 and −20.3 K, respectively. The values of ${\rm{\Delta }}{G}_{{M}_{s}}^{{\gamma }\to {\varepsilon }}$ and ${\rm{\Delta }}{G}_{T}^{{\gamma }\to {\varepsilon }}$ were evaluated at the *M*_*s*_ temperature and at room temperature, respectively, based on the thermodynamic properties used for the SFE calculation (Table 1). Finally, the *τ*_*ε*_ values were calculated using the values of ${\rm{\Delta }}{G}_{{M}_{s}}^{{\gamma }\to {\varepsilon }}$ and ${\rm{\Delta }}{G}_{T}^{{\gamma }\to {\varepsilon }}$, and Eq. (2); final values are listed in Table 2 alongside the CRSS for mechanical twinning (*τ*_*twin*_).

**Table 1:** Physical properties employed for SFE calculation of three utilized steels.

Steel	*μ* (GPa)	*ν*	Lattice parameter (Å)			*d* (μm)	*ρ* (×10^−5^, mol m^−2^)	2*ρΔG*_*ch*_ (mJ m^−2^)	2*ρΔG*_*mag*_ (mJ m^−2^)	2*ρE*_*st*_ (mJ m^−2^)	2*ρΔG*_*AGS*_ (mJ m^−2^)	2*σ* (mJ m^−2^)	SFE (mJ m^−2^)
			austenite	*ε* martensite									
			*a*_*γ*_	*a*_*ε*_	*c*_*ε*_								
0C	70.7	0.279	3.571	2.545*	4.103*	9.9	3.007	−3.7	20.6	1.5	6.0	20.0	44.4
3C	65.4	0.286	3.583	2.548**	4.119**	9.4	2.988	3.7	13.7	0.8	6.1	20.0	44.3
6C	58.0	0.307	3.584	2.548**	4.119**	8.4	2.985	10.6	5.5	0.6	6.5	20.0	43.2

^*^The lattice parameters of *ε* martensite in 0C steel were calculated using empirical equations for parameters of *ε* martensite in binary Fe-Mn steels^41^.

^**^The lattice parameters of *ε* martensite in 3C and 6C steels were taken from parameters of *ε* martensite in Fe-15Mn-0.37C (wt.%) steel^36^.

**Table 2:** Difference in chemical Gibbs free energy between *γ* and *ε* phases at the *ε* martensite start (*M*_*s*_) temperature and at 298 K, and critical resolved shear stresses calculated for both mechanical twinning and *ε* martensitic transformation occurring during plastic deformation in 0C, 3C and 6C steels.

Steel	${\boldsymbol{\Delta }}{{\boldsymbol{G}}}_{{{\boldsymbol{M}}}_{{\boldsymbol{s}}}}^{{\boldsymbol{\gamma }}{\boldsymbol{\to }}{\boldsymbol{\varepsilon }}}\,({\bf{J}}\,{\bf{mo}}{{\bf{l}}}^{{\boldsymbol{-}}1})$	${\boldsymbol{\Delta }}{{\boldsymbol{G}}}_{{\boldsymbol{T}}}^{{\boldsymbol{\gamma }}{\boldsymbol{\to }}{\boldsymbol{\varepsilon }}}\,{\bf{at}}\,{\bf{298}}\,{\rm{K}}\,{\boldsymbol{(}}{\rm{J}}\,{\bf{mo}}{{\bf{l}}}^{{\boldsymbol{-}}1}{\boldsymbol{)}}$	${{\boldsymbol{\tau }}}_{{\boldsymbol{\varepsilon }}}^{{\bf{cal}}{\boldsymbol{.}}}\,({\bf{MPa}})$	${{\boldsymbol{\tau }}}_{{\boldsymbol{twin}}}^{{\bf{cal}}{\boldsymbol{.}}}\,({\bf{MPa}})$
0C	−336.9 ± 110	−62.1	110.0 ± 44	220.5 ± 1
3C	−689.8 ± 110	61.4	300.8 ± 44	211.4 ± 3
6C	−1211.1 ± 110	178.3	556.4 ± 46	196.4 ± 1

Meanwhile, the CRSS for mechanical twinning (*τ*_*twin*_) was evaluated using the following equation proposed by Steinmetz *et al*.^31^. The equation was suggested based on Mahajan and Chin’s twin model^32^ for the creation of a three-layer stacking fault acting as a twin embryo:

3 $${\tau }_{twin}=\frac{{\rm{S}}{\rm{F}}{\rm{E}}}{3{b}_{p}}+\frac{3\mu {b}_{p}}{{L}_{0}}$$

where *b*_*p*_ is the magnitude of the Burgers vector of a partial dislocation ($={a}_{\gamma }/\sqrt{6}$). Using the measured *aγ* value (~0.358 nm) of three steels, the *b*_*p*_ value was calculated and found to be ~0.146 nm. *μ* is the shear modulus and *L*_*0*_ is the width of a twin embryo (260 nm)^31^. The *μ* values of the three steels were measured at room temperature using an ultrasonic pulse-echo measuring system. The *μ* values of the 0C, 3C and 6C steels were 70.7 ± 0.8, 65.4 ± 1.4 and 58.0 ± 0.8 GPa, respectively (Table 1). To evaluate the effects of Mn and C on the *μ* value, the *μ* values of 22 different steels, such as pure Fe, Fe-C, Fe-Mn and Fe-Mn-C steels, measured in previous^15, 33–37^ and present studies, were linearly regressed against the concentrations of Mn and C. Finally, the following equation was obtained as a function of the chemical composition in weight percent:

4 $$\mu ({\rm{GPa}})=(82.63\pm 3.99)-0.35{\rm{Mn}}-12.07{\rm{C}}$$

**Figure 6:**
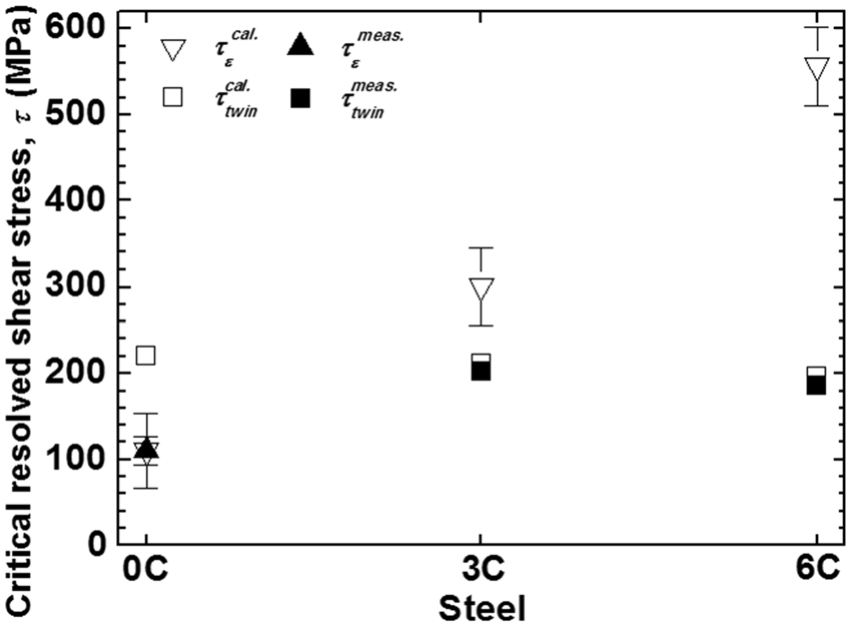
Measured and calculated critical resolved shear stresses for mechanical twinning (*τ*_*twin*_) and for *ε* martensitic transformation (*τ*_*ε*_) in 0C, 3C and 6C steels.

This equation clearly shows that the *μ* value is significantly reduced by the addition of C due to weakened bonding between Fe atoms^37^. Therefore, the lowest *μ* value of the 6C steel is deemed to be caused by high C concentration. Finally, the *τ*_*twin*_ values were calculated using Eq. (3) and the measured *μ* values, as shown in Fig. 6 and Table 2.

Figure 6 shows the calculated and measured values of *τ*_*ε*_ and *τ*_*twin*_. The calculated values came from Eqs (3) and (4); the measured values were obtained using flow curves, critical strains and microstructure. For the measured *τ*_*twin*_ $({{\tau }}_{twin}^{meas.})$, the *σ* value corresponding to the *ε*_*I*_ of the 3C and 6C steels was divided by the Taylor factor of 3.06^38^. For the ${{\tau }}_{{\varepsilon }}^{meas.}$ of 0C steel, because *ε* martensitic transformation started at a strain between 0.05 and 0.10 (Fig. 2b and c), the *σ* values corresponding to *ε* = 0.05 and to *ε* = 0.10 were divided by the Taylor factor of 3.06. Figure 6 clearly shows that the calculated values of *τ*_*ε*_ (${{\tau }}_{{\varepsilon }}^{cal.}$) and *τ*_*twin*_ $({{\tau }}_{twin}^{cal.})$ are in good agreement with the measured values (${{\tau }}_{{\varepsilon }}^{meas.}$ and ${{\tau }}_{twin}^{meas.}$) and that, whereas the *τ*_*ε*_ value is lower than the *τ*_*twin*_ value in the 0C steel, the *τ*_*ε*_ value becomes higher than the *τ*_*twin*_ value in both the 3C and the 6C steels. Namely, while the *τ*_*ε*_ value was greatly increased due to the increase in the difference between the ${\rm{\Delta }}{G}_{{M}_{s}}^{{\gamma }\to {\varepsilon }}$ and ${\rm{\Delta }}{G}_{T}^{{\gamma }\to {\varepsilon }}$ values with increasing C concentration, the *τ*_*twin*_ value was gently reduced due to the slight decrease in *μ* value due to the addition of C. This is the reason that, whilst the 0C steel underwent TRIP, the 3C and 6C steels revealed TWIP.

Meanwhile, although both the 3C and 6C steels exhibited the same deformation mode, *viz*. mechanical twinning, the following differences were observed in their modified C-J plots: (1) the SHR of the 6C steel is higher than that of the 3C steel at all stages. (2) The *ε*_*I*_ value (0.08) of the 6C steel is lower than that (0.13) of the 3C steel. (3) The strain range (0.08–0.50) of stage D in the 6C steel is wider than that (0.13–0.32) of stage D in the 3C steel. These differences in SHR between the 3C and 6C steels result because the 6C steel underwent more active twinning (Figs 3, 4 and 5) due to its lower *τ*_*twin*_ value (Fig. 6), in spite of its similar SFE value and grain size.

In the present study, in order to search for a new design parameter for TWIP steel with an improved combination of strength and ductility, the tensile properties of three austenitic steels (Fe-31Mn, Fe-29Mn-0.3C and Fe-25Mn-0.6C (wt.%)) with the similar SFE (~44 mJ m^−2^) and grain size (~9 μm) were investigated at room temperature. Whereas the 0C steel exhibited *ε* martensitic transformation during tensile deformation, both the 3C and 6C steels showed mechanical twinning. This is because, with increasing C concentration, the *τ*_*twin*_ value for mechanical twinning became lower than the *τ*_*ε*_ value for *ε* martensitic transformation.

Meanwhile, the 6C steel underwent more active mechanical twinning compared to the 3C steel. This is because, due to the decrease in the *μ* value with the increase in C concentration, the 6C steel possessed a lower *τ*_*twin*_ value than that of the 3C steel. These results indicate that both deformation mode and mechanical twinning behavior change with C concentration, although both SFE and grain size are similar. Namely, the addition of C varied the deformation mode from martensitic transformation to mechanical twinning and accelerated the mechanical twinning, resulting in a simultaneous improvement of strength and ductility. Therefore, we realized that for the alloy design of austenitic TWIP steel, not only SFE and grain size but also the values of *μ*, *τ*_*ε*_ and *τ*_*twin*_, which are affected by the chemical composition, particularly the C concentration, should be considered.

## Methods

### Materials selection

For the present study, Fe-31Mn, Fe-29Mn-0.3C and Fe-25Mn-0.6 C (wt.%) steels were used. The three steels had full *γ* austenite phase with similar SFE values of ~44 mJ m^−2^ and average grain size of ~9 μm. The SFE values of the three steels were calculated using a subregular solution model^13^ based on classical nucleation theory^39^:

5 $${\rm{SFE}}\,(\mathrm{mJ}\,{{\rm{m}}}^{-2})=2\rho ({\rm{\Delta }}{G}_{ch}^{{\gamma }\to {\varepsilon }}+{\rm{\Delta }}{G}_{mag}^{{\gamma }\to {\varepsilon }}+{E}_{st}+{\rm{\Delta }}{G}_{AGS})+2{\sigma }$$

where *ρ* (mol m^−2^) is the molar surface density along the atomic plane of (111). ${\rm{\Delta }}{G}_{ch}^{{\gamma }\to {\varepsilon }}$ (J mol^−1^) and ${\rm{\Delta }}{G}_{mag}^{{\gamma }\to {\varepsilon }}$ (J mol^−1^) are the changes of chemical and magnetic Gibbs free energies between the *γ* and *ε* phases, respectively. *E*_*st*_ (J mol^−1^) is the elastic strain energy caused by the difference in specific volume between the *γ* and *ε* phases. Δ*G*_*AGS*_ (J mol^−1^) is the excess free energy introduced by grain refinement of the *γ* phase. *σ* (mJ m^−2^) is the *γ*/*ε* interfacial energy.

The *ρ* value was determined from the measured lattice parameter (*a*_*γ*_) of *γ* austenite, and both ${\rm{\Delta }}{G}_{ch}^{{\gamma }\to {\varepsilon }}$ and ${\rm{\Delta }}{G}_{mag}^{{\gamma }\to {\varepsilon }}$ were calculated using thermodynamic properties adopted for the previous study carried out by the present authors^36^. Meanwhile, the *E*_*st*_ values were calculated according to Eshelby’s inclusion model^40^, which consists of the measurement of dilatational (*E*_*dil*_) and shear (*E*_*sh*_) energies, as follows:

6 $${E}_{st}={E}_{dil}+{E}_{sh}$$

7 $${E}_{dil}=\frac{2(1+{\nu })}{(1-{\nu })}{\mu }{(\frac{1}{3}\frac{{\rm{\Delta }}{V}_{m}^{{\gamma }\to {\varepsilon }}}{{V}_{m}^{{\gamma }}})}^{2}{V}_{m}^{{\gamma }}$$

8 $${E}_{sh}=(\frac{2}{3})\frac{(7-5{\nu })}{15(1-{\nu })}{\mu }{{\varepsilon }}_{33}^{2}{V}_{m}^{{\gamma }}$$

9 $${{\varepsilon }}_{33}=\frac{({c}^{{\varepsilon }}-2{d}_{111}^{{\gamma }})}{2{d}_{111}^{{\gamma }}}$$

where *ν* is the Poisson’s ratio, *μ* is the shear modulus, ${V}_{m}^{{\gamma }}$ and ${V}_{m}^{{\varepsilon }}$ are the molar volumes of the *γ* and *ε* phases, respectively, and ${\rm{\Delta }}{V}_{m}^{{\gamma }\to {\varepsilon }}/{V}_{m}^{{\gamma }}$ is the volume change due to the *γ* to *ε* martensitic transformation. *ε*_33_ is a normal strain to a faulted plane along the *c*-axis, *c*_*ε*_ is the *c* lattice parameter of the *ε* phase, and ${d}_{111}^{\gamma }$ is the distance between (111) atomic planes in the *γ* phase^13, 15^.

The *E*_*st*_ values of the three steels were calculated using the measured elastic moduli (*ν* and *μ*), the lattice parameters (*a*_*γ*_, *a*_*ε*_ and *c*_*ε*_) of the *γ* and *ε* phases and Eqs (6–9). Because the three undeformed steels had a *γ* single phase at room temperature, the *a*_*ε*_ and *c*_*ε*_ values of the Fe-31Mn (wt.%) steel were calculated using empirical equations for binary Fe-Mn steels^41^. The *a*_*ε*_ and *c*_*ε*_ values of the Fe-29Mn-0.3C and Fe-25Mn-0.6C (wt.%) steels were determined to be the *a*_*ε*_ and *c*_*ε*_ values measured using Fe-15Mn-0.37C (wt.%) steel^36^.

Regarding Δ*G*_*AGS*_, the following equation proposed by Lee and Choi^42^ was employed: Δ*G*_*AGS*_ (J mol^−1^) = 170.06 exp(−*d*/18.55). Here, *d* (μm) is the average grain size of austenite. A constant value of 10 mJ m^−2^ was employed for the *σ* value. The various energies and physical parameters used for the thermodynamic calculation of SFE are listed in Table 1.

### Materials preparation

Using a high frequency vacuum induction furnace, Fe-31Mn, Fe-29Mn-0.3C and Fe-25Mn-0.6C (wt.%) steels were first made as 30 kg ingots. The ingots were homogenized at 1200 °C for 12 h, hot-rolled to 6-mm-thick plates at ~1100 °C, and then air cooled to room temperature. The actual chemical compositions of the three steels were analyzed using the hot-rolled plates; results are listed in Supplementary Table 1.

After each side of the hot-rolled plates was ground 1 mm, the plates were cold-rolled at room temperature to make 2.4-mm-thick sheets, corresponding to a thickness reduction of 40%. The cold-rolled sheets were subsequently annealed at 900 °C for 10 min using a tube furnace under a vacuum condition of ~5 × 10^−2^ torr; this was followed by water quenching to prevent carbide precipitation during cooling.

### SEM and EBSD analysis

The constituent phases and mechanical twins of both the annealed and tensile-deformed specimens were observed using a field-emission scanning electron microscope (FE-SEM; JEOL, JSM-7001F), operated at 20 kV and equipped with an electron backscatter diffractometer (EBSD; Hikari, EDAX-TSL). The step size and the working distance for the EBSD analysis were 0.09 μm and 14 mm, respectively. The surfaces of the EBSD specimens were polished using a suspension including 0.04-μm colloidal silica particles; using an electro-polisher (Struers, LectroPol-5), surfaces were then electrochemically polished at 15 °C for 30 s in a mixed solution of 90% glacial acetic acid (CH_3_COOH) and 10% perchloric acid (HClO_4_) to remove the mechanically damaged layer. Using point counting analysis of EBSD image quality maps^17, 43^, the fraction of mechanical twins was measured as a function of the tensile strain.

### Mechanical testing

Tensile specimens with a gauge portion measuring 25 mm in length, 6 mm in width and 2.4 mm in thickness, corresponding to the ASTM E 8M-04 sub-size, were machined along the rolling direction from the cold-rolled sheets. Annealed tensile specimens were mechanically ground, electro-polished and then deformed at an initial strain rate of 1 × 10^−4^ s^−1^ at room temperature using an Instron 3382 machine.

### X-ray diffraction

The *a*_*γ*_ value of the annealed specimen was examined using an X-ray diffractometer (XRD; Rigaku, D/MAX-RINT 2700) with Cu-Kα radiation (λ = 1.5405 Å). The XRD was operated at room temperature with a scanning range from 40 to 100°, a scanning rate of 0.5° min^−1^ and a step size of 0.01°. The *a*_*γ*_ value was calculated from all diffracted peaks of the *γ* phase and then averaged^44^.

### Measurement of elastic constants

For the accurate evaluation of the *E*_*st*_ value and the critical resolved shear stress for mechanical twinning, both the Young’s and the shear moduli of annealed specimens were measured at room temperature using an ultrasonic pulse-echo measuring system (HKLAB CO., HKL-01-UEMT). The room-temperature Poisson’s ratios of the annealed specimens were calculated using an elastic equation for isotropic bodies^45^ and the measured Young’s and shear moduli.

## Electronic supplementary material

Supplementary information
