# Supplementary material for: Design for Fe-high Mn alloy with an improved combination of strength and ductility
Source: Sci Rep. 2017 Jun 15;7:3573. doi: 10.1038/s41598-017-03862-y (PMC5472627; doi:10.1038/s41598-017-03862-y)
Supplement: Supplementary file 1 — Supplementary information [file 41598_2017_3862_MOESM1_ESM.doc]

**Design for Fe-high Mn alloy with an improved combination of strength and ductility**

Seung-Joon Lee^1,a^, Jeongho Han^1,b^, Sukjin Lee^1^, Seok-Hyeon Kang^1^, Sang-Min Lee^1^ and Young-Kook Lee^1,*^

*^1^Department of Materials Science and Engineering, Yonsei University, Seoul 03722, Republic of Korea*

*^a^Current affiliation*: *Joining and Welding Research Institute, Osaka University, 11-1, Osaka 567-0047, Japan*

*^b^Current affiliation: Department of Materials Science and Engineering, Chungnam National University, Daejeon 34134, Republic of Korea*

^*^Corresponding author: E-mail: yklee@yonsei.ac.kr, Phone: +82 2 2123 2831

**Supplementary Table 1. Chemical compositions (wt.%) of three Fe-high Mn steels used in the present study.**

| Steel | C | Mn | S | Fe |
| --- | --- | --- | --- | --- |
| 0C | 0.002 | 31.1 | 0.005 | bal. |
| 3C | 0.270 | 28.5 | 0.009 | bal. |
| 6C | 0.620 | 24.5 | 0.017 | bal. |

**Supplementary Table 2. Room-temperature tensile properties of 0C, 3C and 6C steels annealed at 900 °C for 10 min.**

| Steel | Yield strength (MPa) | Ultimate tensile strength (MPa) | Total elongation (%) |
| --- | --- | --- | --- |
| 0C | 160 | 532 | 60 |
| 3C | 258 | 797 | 67 |
| 6C | 312 | 1000 | 79 |
